# Iodine-mediated one-pot intramolecular decarboxylation domino reaction for accessing functionalised 2-(1,3,4-oxadiazol-2-yl)anilines with carbonic anhydrase inhibitory action

**DOI:** 10.1080/14756366.2018.1443447

**Published:** 2018-03-14

**Authors:** Srinivas Angapelly, P. V. Sri Ramya, Rohini Sodhi, Andrea Angeli, Krishnan Rangan, Narayana Nagesh, Claudiu T. Supuran, Mohammed Arifuddin

**Affiliations:** a National Institute of Pharmaceutical Education and Research (NIPER) – Hyderabad, Hyderabad, India;; b Neurofarba Department, Sezione di Scienze Farmaceutiche e Nutraceutiche, University of Florence, Florence, Italy;; c Birla Institute of Technology & Science, Pilani, Hyderabad, India;; d Center for Cellular and Molecular Biology (CCMB), Hyderabad, India

**Keywords:** Domino synthesis, 1,3,4-oxadiazole, carbonic anhydrase, sulphonamide, iodine

## Abstract

A practical and transition metal-free one-pot domino synthesis of diversified (1,3,4-oxadiazol-2-yl)anilines has been developed employing isatins and hydrazides as the starting materials, in the presence of molecular iodine. The prominent feature of this domino process involves consecutive condensation, hydrolytic ring cleavage, and an intramolecular decarboxylation, in a one-pot process that leads to the oxidative formation of a C–O bond. Fluorescence properties of some of the representative molecules obtained in this way were studied. The synthesised 2-(1,3,4-oxadiazolo-2-yl)aniline-benzene sulphonamides (**8a**–**o)** were screened for their carbonic anhydrase (CA, EC 4.2.1.1) inhibitory activity. Most of the compounds exhibited low micromolar to nanomolar activity against human (h) isoforms hCA I, hCA II, hCA IV, and XII, with some compounds displaying selective CA inhibitory activity towards hCA II with K_I_s of 6.4–17.6 nM.

## Introduction

**Scheme 1.**
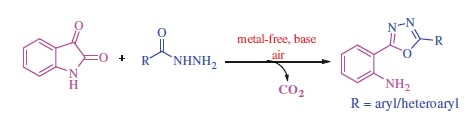
Transition metal-free domino oxidative decarboxylation for the formation of 1,3,4-oxadiazole.

Construction of *O*–heterocyclic ring systems via intramolecular C–O bond formation has become an emerging tool in drug discovery. Accordingly, many efforts have been devoted to this activity, and remarkable results have been achieved to date. Among these, the traditional intramolecular Pd-catalysed Hartwig–Buchwald^1^ and copper-catalysed^2^ Ullmann-type C–O coupling of aryl halides with hydroxyl moieties, and in an alternative approach, the direct dehydrogenative coupling occurs between C–H and O–H bonds^3^, leading to various functionalised compounds. In most cases, these elaborative designs implied complex catalytic systems (based on Pd(II), Cu(II), Rh(III), and Ru(III) derivatives) and multi-step processes for the preparation of diversely functionalised derivatives, such as, furan, pyrrole, pyrazole, isoquinoline, indole, benzoxazole, and carbazole ring systems^4^. However, oxidative decarboxylation leading to construction of *C*–heteroatom bonds, particularly the C–O and the C–N bonds, has received significantly less attention. In recent years, in the perspective of green chemistry, most of the organic chemists have switched to metal-free reactions to reduce the burden of toxicity. In this context, iodine and hypervalent iodine reagents have emerged as inexpensive, versatile, and environmentally more friendly reagents^5^. Structural features and the reactivity pattern of these iodine compounds in many aspects are similar to those of the transition metal compounds applied for such purposes. Up until now, many efforts have been made to directly functionalise C–H bonds for the construction of C–C and *C*–heteroatoms bonds by employing iodine or hypervalent iodine reagents^6^^,^^7^. Wang et al. demonstrated a facile access to various heterocycles (quinazoline, oxazole, and pyridine) through the tandem oxidative coupling reactions using iodine as catalyst and *tert*-butyl-hydroperoxide (TBHP) as the oxidant^8^. Furthermore, Ma et al. proposed the synthesis of imidazo[1,2-*a*]pyridines via oxidative coupling of 2-aminopyridine with 1,3-diketones in the presence of tetra-butylammonium iodide (TBAI), TBHP, and BF_3_·etherate^9^. Very recently Tang et al. reported iodine-catalysed radical oxidative annulation for the synthesis of dihydrofurans and indolisines^10^. Interestingly, I_2_ (or hypervalent iodine derivatives) also promoted the oxidative decarboxylation of amino acids and β,γ-unsaturated carboxylic acids^11^. Intrigued by these advances, herein we envisioned a metal-free, iodine-mediated domino strategy involving intramolecular decarboxylative coupling of isatins, and hydrazides for the synthesis of 2-(1,3,4-oxadiazol-2-yl)aniline derivatives (Scheme 1).

**Figure 1.**
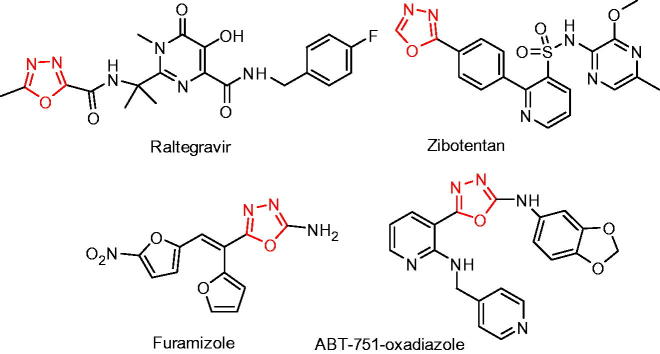
Some of the bioactive compounds containing 1,3,4-oxadiazole moiety.

1,3,4-Oxadiazole motif is an important five-membered aromatic heterocyclic ring present in many bioactive molecules^12^^,^^13^, including anticancer, antibacterial, anti-inflammatory, anti-diabetic, antiviral, anticonvulsant, analgesic, and antifungal agents^12^^,^^13^. Some of the drugs and drug candidates, such as raltegravir, zibotentan, furamizole, and ABT-751-oxadiazole possessing 1,3,4-oxadiazole moieties are depicted in Figure 1. Apart from biology, their applications have also been extended to material chemistry due to their unique optoelectronic properties^14^.

To date, a number of synthetic protocols have been described in the literature to access 1,3,4-oxadiazoles^15–17^. They include: (i) oxidative cyclisation of *N*-acylhydrazones with FeCl_3_, CAN, PbO_2_, hypervalent iodines, chloramine T, KMnO_4_, Br_2_, HgO/I_2_; (ii) From 1,2-diacylhydrazones via cyclodehydration by employing PPA, POCl_3_, SOCl_2_, and H_2_SO_4_; (iii) Arylation of preformed 2-substituted 1,3,4-oxadiazoles through C–H activation^18^. Additionally, Guin et al. successfully accomplished 2,5-disubstituted 1,3,4-oxadiazoles from *N*-arylidenearoyl hydrazides using Cu(OTf)_2_^19^. Recently, Xu et al. demonstrated an easy access to synthesise 2-(1,3,4-oxadiazol-2-yl)anilines by employing CuI as the catalyst^20^. Nevertheless, the problems associated with these protocols, including the use of expensive, hazardous materials, or inefficient multi-step processes endowed them with a limited applicability. Therefore, more general and eco-friendly strategies for the synthesis of functionalised 1,3,4-oxadiazoles from easily available starting materials are still highly desirable. This prompted us to explore a simpler and more efficient protocol which is reported in this article.

## Materials and methods

### Chemistry

All solvents were purified and dried using standard methods prior to use. Commercially available reagents were used without further purification. All reactions involving air- or moisture-sensitive compounds were performed under a nitrogen atmosphere using dried glassware and syringe techniques to transfer solutions. Analytical thin-layer chromatography (TLC) was carried out on Merck silica gel 60 F-254 aluminium plates. Melting points were determined on Stuart digital melting-point apparatus/SMP 30 in open capillary tubes and uncorrected. Nuclear magnetic resonance (^1^H-NMR, ^13 ^C-NMR) spectra were recorded using an Avance Bruker 500 MHz, 125 MHz spectrometer in DMSO-d_6_. Chemical shifts reported in parts per million (ppm) with TMS as an internal reference, and the coupling constants (*J*) expressed in hertz (Hz). Splitting patterns are denoted as follows: s, singlet; d, doublet; t, triplet; m, multiplet; dd, doublet of doublet. HRMS were determined with Agilent QTOF mass spectrometer 6540 series instrument and were performed in the ESI techniques at 70 eV.

#### General procedure for the preparation of 2–(1,3,4-oxadiazol-2-yl) aniline derivatives (3a–u), (6a–g), and (8a–o)

A glass tube charged with a mixture of the desired isatin (0.5 mmol), aryl or heteroaryl hydrazide (0.5 mmol), I_2_ (100 mol%), K_2_CO_3_ (1.5 equiv.), and then 3 ml of DMSO at room temperature, was sealed and the resulting mixture was stirred under microwave irradiation at 160 °C until the disappearance of the reactants (monitored by TLC in 20% EtOAc and hexane). Iodine was then quenched by the addition of 10% aqueous Na_2_S_2_O_3_ and the product was extracted with EtOAc (3 × 25 ml). The combined extract was washed with brine, dried over anhydrous Na_2_SO_4_ and was concentrated under reduced pressure. The residue was purified by column chromatography on 60–120 silica gel using a mixture of EtOAc (bp 77 °C) and petroleum ether (bp 42–60 °C) as eluent to afford the desired product (correspondingly, **3a**–**3u**/**6a**–**6g/8a–o**) as a yellow solid (yield, 69–92%).

#### 2-(5-(p-Tolyl)-1,3,4-oxadiazol-2-yl)aniline (3a)

Yellow solid, Yield: 114 mg (91%), m.p 174–175 °C. ^1^H NMR (500 MHz, CDCl_3_) δ 8.02 (d, *J* = 7.7 Hz, 2H), 7.85 (d, *J* = 7.6 Hz, 1H), 7.31 (dd, *J* = 23.7, 7.5 Hz, 3H), 6.79 (dd, *J* = 14.7, 7.7 Hz, 2H), 5.89 (s, 2H), and 2.44 (s, 3H). ^13 ^C NMR (126 MHz, DMSO) δ 164.5, 162.6, 148.2, 142.6, 132.9, 130.4, 128.2, 127.0, 120.9, 116.3, 116.1, 104.5, and 21.6. HRMS (ESI) *m/z* [M + H]^+^ calculated for C_15_H_14_N_3_O 252.1131, found 252.1136.

#### 2-(5-(4-Methoxy-2-methylphenyl)-1,3,4-oxadiazol-2-yl)aniline (3b)

Yellow solid, Yield: 126 mg (90%), m.p 148.5–150 °C. ^1^H NMR (500 MHz, DMSO) δ 8.02 (d, *J* = 8.7 Hz, 1H), 7.80 (dd, *J* = 8.0, 1.4 Hz, 1H), 7.31–7.25 (m, 1H), 7.03 (d, *J* = 2.4 Hz, 1H), 6.99 (dd, *J* = 8.7, 2.6 Hz, 1H), 6.92 (d, *J* = 8.3 Hz, 1H), 6.78 (s, 2H), 6.73–6.68 (m, 1H), 3.84 (s, 3H), and 2.68 (s, 3H). ^13 ^C NMR (126 MHz, DMSO) δ 163.8, 162.7, 161.8, 148.2, 140.30, 132.80, 131.13, 128.1, 117.3, 116.3, 116.0, 115.3, 112.6, 104.6, 55.8, and 22.3. HRMS (ESI) *m/z*: [M + H]^+^ calculated for C_16_H_16_N_3_O_2_ 282.1237, found 282.1240.

#### 2-(5-(3,4,5-Trimethoxyphenyl)-1,3,4-oxadiazol-2-yl)aniline (3c)

Yellow solid, Yield: 149 mg (91%), m.p 169–171 °C. ^1^H NMR (500 MHz, DMSO) δ 7.94 (dd, *J* = 8.0, 1.4 Hz, 1H), 7.39 (s, 2H), 7.30 (ddd, *J* = 8.5, 7.2, 1.5 Hz, 1H), 6.93 (dd, *J* = 8.3, 0.6 Hz, 1H), 6.79 (s, 2H), 6.75–6.69 (ddd,.1H), 3.92 (s, 6H), 3.77 (s, 3H). ^13 ^C NMR (126 MHz, DMSO) δ 164.7, 162.4, 153.9, 148.3, 141.0, 133.0, 128.4, 118.9, 116.3, 115.9, 104.5, 104.4, 60.7, and 56.6. HRMS (ESI) *m/z*: [M + H]^+^ calculated for C_17_H_18_F_3_N_3_O_4_ 328.1292, found 328.1291.

#### 2-(5-(4-(Trifluoromethyl)phenyl)-1,3,4-oxadiazol-2-yl)aniline (3d)

Yellow solid, Yield: 129 mg (84%), m.p 195–197 °C. ^1^H NMR (500 MHz, DMSO) δ 8.34 (d, *J* = 8.0 Hz, 2H), 7.98 (d, *J* = 8.0 Hz, 2H), 7.88 (d, *J* = 7.8 Hz, 1H), 7.30 (t, *J* = 7.5 Hz, 1H), 6.93 (d, *J* = 8.3 Hz, 1H), 6.80 (s, 2H), and 6.72 (t, *J* = 7.4 Hz, 1H). ^13 ^C NMR (126 MHz, DMSO) δ 165.3, 161.4, 148.5, 133.2, 128.3, 127.9, 126.7 (d, *J* = 3.7 Hz), 125.3, 123.1, 116.4, 116.0, and 104.1. HRMS (ESI) *m/z*: [M + H]^+^ calculated for C_15_H_11_F_3_N_3_O 306.0849, found 306.0846.

#### 2-(5-(4-Fluorophenyl)-1,3,4-oxadiazol-2-yl)aniline (3e)

Yellow solid, Yield: 106 mg (83%), m.p 170–172 °C. ^1^H NMR (500 MHz, CDCl_3_) δ 8.21–8.10 (m, 2H), 7.84 (dd, *J* = 7.9, 1.3 Hz, 1H), 7.32–7.27 (m, 1H), 7.26–7.19 (m, 2H), 6.80 (dd, *J* = 15.8, 7.8 Hz, 2H), and 5.83 (s, 2H). ^13 ^C NMR (126 MHz, CDCl_3_) δ 165.7, 163.77, 162.00, 147.1, 132.6, 129.20 (d, *J* = 8.9 Hz), 127.7, 120.24 (d, *J* = 3.3 Hz), 116.8, 116.5, 116.3, and 105.61. HRMS (ESI) *m/z*: [M + H]^+^ calculated for C_14_H_11_FN_3_O 256.0881, found 256.0878.

#### 2-(5-(4-Bbromophenyl)-1,3,4-oxadiazol-2-yl)aniline (3f)

Yellow solid, Yield: 134 mg (85%), m.p 186–187 °C. ^1^H NMR (500 MHz, DMSO) δ 8.10–8.06 (m, 2H), 7.89–7.82 (m, 3H), 7.30 (ddd, *J* = 8.5, 7.2, 1.5 Hz, 1H), 6.93 (d, *J* = 8.3 Hz, 1H), 6.79 (d, *J* = 7.3 Hz, 2H), and 6.74–6.69 (m, 1H). ^13 ^C NMR (126 MHz, DMSO) δ 164.9, 161.8, 148.4, 133.1, 132.9, 129.0, 128.3, 125.9, 123.0, 116.4, 116.0, and 104.3. HRMS (ESI) *m/z*: [M + H]^+^ calculated for C_14_H_11_BrN_3_O 316.0080, found 316.0078.

#### 2-(5-(3,5-Dichlorophenyl)-1,3,4-oxadiazol-2-yl)aniline (3g)

Yellow solid, Yield: 126 mg (83%), m.p 210–212 °C. ^1^H NMR (500 MHz, DMSO) δ 8.16 (d, *J* = 1.9 Hz, 2H), 7.98 (dd, *J* = 8.0, 1.5 Hz, 1H), 7.92 (t, *J* = 1.9 Hz, 1H), 7.30 (ddd, *J* = 8.5, 7.1, 1.6 Hz, 1H), 6.92 (dd, *J* = 8.4, 0.7 Hz, 1H), 6.79 (s, 2H), and 6.73–6.68 (m, 1H). ^13 ^C NMR (126 MHz, DMSO) δ 165.4, 160.5, 148.5, 135.6, 133.3, 131.64, 128.7, 127.0, 125.5, 116.3, 116.0, and 104.0. HRMS (ESI) *m/z* [M + H]^+^ calculated for C_14_H_10_Cl_2_N_3_O 306.0915, found 306.0918.

#### 2-(5-(2-Methyl-5-nitrophenyl)-1,3,4-oxadiazol-2-yl)aniline (3i)

Yellow solid, Yield: 120 mg (81%), m.p 186.5–188 °C. ^1^H NMR (500 MHz, DMSO) δ 8.12 (d, *J* = 8.3 Hz, 1H), 8.00–7.96 (m, 1H), 7.73 (d, *J* = 8.2 Hz, 1H), 7.65 (dd, *J* = 8.0, 1.3 Hz, 1H), 7.33–7.27 (m, 1H), 6.94 (d, *J* = 8.3 Hz, 1H), 6.80 (s, 2H), 6.73–6.66 (m, 1H), 2.52 (s, 3H). ^13 ^C NMR (126 MHz, DMSO) δ 165.4, 159.3, 148.4, 146.2, 145.3, 133.9, 133.4, 132.0, 128.0, 125.3, 117.6, 116.5, 116.1, 103.8, and 21.2. HRMS (ESI) *m/z*: [M + H]^+^ calculated for C_15_H_13_N_4_O_3_ 297.0982, found 297.0976.

#### 4-Chloro-2-(5-(4-(trifluoromethyl)phenyl)-1,3,4-oxadiazol-2-yl)aniline (3j)

Yellow solid, Yield: 137 mg (81%), m.p 196–198 °C. ^1^H NMR (500 MHz, DMSO) δ 8.34 (t, *J* = 44.9 Hz, 2H), 8.17–7.76 (m, 3H), 7.32 (d, *J* = 7.2 Hz, 1H), 6.95 (s, 3H). ^13 ^C NMR (126 MHz, DMSO) δ 164.2, 161.8, 147.3, 132.9, 128.16, 127.45, 127.19, 126.7 (d, *J* = 3.7 Hz), 125.33, 119.26, 118.3, and 105.1. HRMS (ESI) *m/z*: [M + H]^+^calculated for C_15_H_10_ClF_3_N_3_O 340.0459, found 340.0462.

#### 4-Chloro-2-(5-(4-fluorophenyl)-1,3,4-oxadiazol-2-yl)aniline (3k)

Yellow solid, Yield: 117 mg (81%), m.p 176–177.5 °C. ^1^H NMR (500 MHz, DMSO) δ 8.29–8.23 (m, 2H), 7.91 (d, *J* = 2.5 Hz, 1H), 7.50–7.44 (m, 2H), 7.31 (dd, *J* = 8.9, 2.5 Hz, 1H), 6.96 (d, *J* = 8.9 Hz, 1H), 6.92 (s, 2H). ^13 ^C NMR (126 MHz, DMSO) δ 165.67, 163.78, 162.0, 147.1, 132.7, 130.0 (d, *J* = 9.1 Hz), 127.1, 120.0 (d, *J* = 3.1 Hz), 119.2, 118.2, 117.1, 116.9, and 105.4. HRMS (ESI) *m/z*: [M + H]^+^ calculated for C_14_H_10_ClFN_3_O 290.0941, found 290.0939.

#### 2-(5-(4-Bromophenyl)-1,3,4-oxadiazol-2-yl)-4-chloroaniline (3l)

Yellow solid, Yield: 145 mg (83%), m.p 196–197 °C. ^1^H NMR (500 MHz, DMSO) δ 8.17–8.13 (m, 2H), 7.93 (d, *J* = 2.5 Hz, 1H), 7.87–7.82 (m, 2H), 7.33 (dd, *J* = 8.9, 2.5 Hz, 1H), 6.97–6.91 (m, 3H). ^13 ^C NMR (126 MHz, DMSO) δ 163.9, 162.2, 147.2, 132.9, 132.8, 129.2, 127.1, 126.1, 122.9, 119.2, 118.2, and 105.3. HRMS (ESI) *m/z*: [M + H]^+^ calculated for C_14_H_10_BrClN_3_O 349.9690, found 349.9698.

#### 4-Chloro-2-(5-(3,5-dichlorophenyl)-1,3,4-oxadiazol-2-yl)aniline (3m)

Yellow solid, Yield: 137 mg (81%), m.p 229–231 °C. ^1^H NMR (500 MHz, DMSO) δ 8.24 (d, *J* = 1.8 Hz, 2H), 8.06 (d, *J* = 2.5 Hz, 1H), 7.92 (t, *J* = 1.8 Hz, 1H), 7.32 (dd, *J* = 8.9, 2.5 Hz, 1H), 6.97–6.92 (m, 3H). ^13 ^C NMR (126 MHz, DMSO) δ 164.4, 160.8, 147.3, 135.6, 133.06, 131.7, 127.4, 126.8, 125.75, 119.35, 118.2, and 105.06. HRMS (ESI) *m/z*: [M + H]^+^ calculated for C_14_H_9_Cl_3_N_3_O 339.9806, found 339.9811.

#### 4-Chloro-2-(5-(5-methoxy-2-methylphenyl)-1,3,4-oxadiazol-2-yl)aniline (3n)

Yellow solid, Yield: 140 mg (89%), m.p 158–15.5 °C. ^1^H NMR (500 MHz, DMSO) δ 8.08 (d, *J* = 8.7 Hz, 1H), 7.79 (d, *J* = 2.4 Hz, 1H), 7.30 (dd, *J* = 8.9, 2.5 Hz, 1H), 7.02 (d, *J* = 2.1 Hz, 1H), 6.99–6.90 (m, 4H), 3.84 (s, 3H), and 2.67 (s, 3H). ^13 ^C NMR (126 MHz, DMSO) δ 162.9, 162.74, 161.8, 147.0, 140.4, 132.4, 131.3, 126.9, 119.1, 118.1, 117.3, 115.1, 112.6, 105.6, 55.87, and 22.3. HRMS (ESI) *m/z*: [M + H]^+^ calculated for C_16_H_15_ClN_3_O_2_ 316.0847, found 316.0844.

#### 4-Bromo-2-(5-(4-(trifluoromethyl)phenyl)-1,3,4-oxadiazol-2-yl)aniline (3o)

Yellow solid, Yield: 157 mg (82%), m.p 167–168 °C. ^1^H NMR (500 MHz, DMSO) δ 8.40 (d, *J* = 7.4 Hz, 2H), 8.09–7.93 (m, 3H), 7.42 (d, *J* = 8.4 Hz, 1H), 6.97 (s, 2H), 6.91 (d, *J* = 8.8 Hz, 1H). ^13 ^C NMR (126 MHz, DMSO) δ 164.2, 161.8, 147.6, 135.6, 130.0, 128.1, 127.4, 126.74 (d, *J* = 3.7 Hz), 125.3, 118.6, 106.2, and 105.85. HRMS (ESI) *m/z*: [M + H]^+^ calculated for C_15_H_10_BrF_3_N_3_O 383.9984, found 383.9990.

#### 2-(5-(3,5-Dimethoxyphenyl)-1,3,4-oxadiazol-2-yl)-4-fluoroaniline (3p)

Yellow solid, Yield: 139 mg (88%), m.p 152–154 °C. ^1^H NMR (500 MHz, DMSO) δ 7.78 (dd, *J* = 9.6, 3.0 Hz, 1H), 7.28 (t, *J* = 8.4 Hz, 2H), 7.21 (td, *J* = 8.7, 3.0 Hz, 1H), 6.94 (dd, *J* = 9.1, 4.8 Hz, 1H), 6.76 (t, *J* = 2.2 Hz, 1H), 6.70 (s, 2H), 3.87 (s, 6H). ^13 ^C NMR (126 MHz, DMSO) δ 164.1 (d, *J* = 2.6 Hz), 162.6, 161.4, 154.5, 152.7, 145.2, 125.2, 120.8 (d, *J* = 22.5 Hz), 117.8 (d, *J* = 7.4 Hz), 113.4 (d, *J* = 24.4 Hz), 105.4, 104.4, 104.01 (d, *J* = 8.2 Hz), and 56.11 (s). HRMS (ESI) *m/z*: [M + H]^+^ calculated for C_16_H_15_FN_3_O_3_ 316.1092, found 316.1094.

#### 2-(5-(4-Fluorophenyl)-1,3,4-oxadiazol-2-yl)-4-(trifluoromethoxy)aniline (3q)

Yellow solid, Yield: 129 mg (80%), m.p 183–184 °C. ^1^H NMR (500 MHz, DMSO) δ 8.30–8.23 (m, 18H), 7.87 (d, *J* = 2.6 Hz, 8H), 7.48 (t, *J* = 8.8 Hz, 18H), 7.32 (dd, *J* = 9.0, 2.0 Hz, 9H), 7.02 (s, 5H), 7.00 (s, 17H). ^13 ^C NMR (126 MHz, DMSO) δ 165.6, 163.7 (d, *J* = 9.4 Hz), 162.1, 147.5, 138.1 (d, *J* = 2.0 Hz), 130.1 (d, *J* = 9.2 Hz), 126.5, 121.8, 120.9, 120.3 (d, *J* = 3.0 Hz), 119.8, 117.6, 117.1 (d, *J* = 22.5 Hz), and 104.2. HRMS (ESI) *m/z*: [M + H]^+^ calculated for C_15_H_10_F_4_N_3_O_2_ 340.0755, found 340.0759.

#### 4-(Trifluoromethoxy)-2-(5-(3,4,5-trimethoxyphenyl)-1,3,4-oxadiazol-2-yl)aniline (3r)

Yellow solid, Yield: 172 mg (87%), m.p 203.5–205 °C. ^1^H NMR (500 MHz, DMSO) δ 7.93 (s, 1H), 7.44 (s, 2H), 7.32 (s, 1H), 7.01 (s, 3H), 3.93 (s, 6H), 3.77 (s, 3H). ^13 ^C NMR (126 MHz, DMSO) δ 163.7, 162.8, 153.9, 147.4, 141.2, 138.1, 126.4, 121.0, 118.7, 117.6, 104.9, 104.2, 60.7, and 56.8. HRMS (ESI) *m/z*: [M + H]^+^ calculated for C_18_H_17_F_3_N_3_O_5_ 411.1166, found 411.1169.

#### 2-(5-(3,5-Dimethylphenyl)-1,3,4-oxadiazol-2-yl)-4-(trifluoromethoxy)aniline (3s)

Yellow solid, Yield: 144 mg (84%), m.p 199–201 °C. ^1^H NMR (500 MHz, DMSO) δ 7.78 (dd, *J* = 9.6, 3.0 Hz, 1H), 7.28 (t, *J* = 8.4 Hz, 2H), 7.21 (td, *J* = 8.7, 3.0 Hz, 1H), 6.94 (dd, *J* = 9.1, 4.8 Hz, 1H), 6.76 (t, *J* = 2.2 Hz, 1H), 6.70 (s, 2H), 3.87 (s, 6H). ^13 ^C NMR (126 MHz, DMSO) δ 163.6, 163, 147.4, 139.2, 138, 133, 126.4, 124.8, 123.4, 120.9, 117.6, 104.3, and 21.1. HRMS (ESI) *m/z*: [M + H]^+^ calculated for C_17_H_15_F_3_N_3_O 350.1162, found 350.1158.

#### 2-(5-(5-Methoxy-2-methylphenyl)-1,3,4-oxadiazol-2-yl)-4-nitroaniline (3t)

Yellow solid, Yield: 139 mg (85%), m.p 240–241 °C. ^1^H NMR (500 MHz, DMSO) δ 8.63 (t, *J* = 9.8 Hz, 1H), 8.21–7.92 (m, 4H), 7.10–6.99 (m, 3H), 3.84 (d, *J* = 20.0 Hz, 3H), 2.67 (d, *J* = 25.2 Hz, 3H). ^13 ^C NMR (126 MHz, DMSO) δ 163.3, 162.2, 162.0, 153.0, 140.5, 136.3, 131.4, 128.0, 125.4, 117.3, 116.2, 114.9, 112.7, 103.9, 55.9, and 22.3. HRMS (ESI) *m/z*: [M + H]^+^ calculated for C_16_H_15_N_4_O_4_ 327.1088, found 327.1089.

#### 2-(5-(3,5-Dimethoxyphenyl)-1,3,4-oxadiazol-2-yl)-4-methoxyaniline (3u)

Yellow solid, Yield: 150 mg (92%), m.p 143.5–145 °C. ^1^H NMR (500 MHz, DMSO) δ 8.30–8.23 (m, 18H), 7.87 (d, *J* = 2.6 Hz, 8H), 7.48 (t, *J* = 8.8 Hz, 18H), 7.32 (dd, *J* = 9.0, 2.0 Hz, 9H), 7.02 (s, 5H), and 7.00 (s, 17H). ^13 ^C NMR (126 MHz, DMSO) δ 164.80, 162.41, 161.47, 150.35, 143.13, 125.36, 121.72, 118.03, 111.04, 105.08, 104.13, 104.12, 56.19, and 56.08. HRMS (ESI) *m/z*: [M + H]^+^ calculated for C_17_H_18_N_3_O_4_ 328.1292, found 328.1290.

#### 2-(5-(Pyridin-4-yl)-1,3,4-oxadiazol-2-yl)aniline (6a)

Yellow solid, Yield: 108 mg (90%), m.p 174–175 °C. ^1^H NMR (500 MHz, DMSO) δ 8.85 (dd, *J* = 4.5, 1.6 Hz, 2H), 8.07 (dd, *J* = 4.5, 1.5 Hz, 2H), 7.89 (dd, *J* = 8.0, 1.4 Hz, 1H), 7.31 (ddd, *J* = 8.5, 7.2, 1.5 Hz, 1H), 6.94 (d, *J* = 7.9 Hz, 1H), 6.81 (s, 2H), 6.76–6.68 (m, 1H). ^13 ^C NMR (126 MHz, DMSO) δ 165.5, 161.0, 151.3, 148.6, 133.4, 130.9, 128.4, 120.7, 116.5, 116.0, and 104.0. HRMS (ESI) *m/z*: [M + H]^+^ calculated for C_13_H_11_N_4_O 239.0927, found 239.0928.

#### 2-(5-(Isoquinolin-3-yl)-1,3,4-oxadiazol-2-yl)aniline (6b)

Yellow solid, Yield: 126 mg (87%), m.p 256–257 °C. ^1^H NMR (500 MHz, CDCl_3_) δ 9.43 (s, 1H), 8.69 (s, 1H), 8.13–7.96 (m, 3H), 7.83 (t, *J* = 7.4 Hz, 1H), 7.76 (t, *J* = 7.3 Hz, 1H), 7.35–7.27 (m, 1H), 6.86–6.79 (m, 2H). ^13 ^C NMR (126 MHz, DMSO) δ 165.20, 162.55, 153.88, 148.50, 136.96, 135.62, 133.17, 132.25, 130.01, 129.37, 128.43, 128.2, 128.1, 121.16, 116.50, 116.11, and 104.40. HRMS (ESI) *m/z*: [M + H]^+^ calculated for C_17_H_13_N_4_O 289.1084, found 289.1088.

#### 2-(5-(1H-indazol-3-yl)-1,3,4-oxadiazol-2-yl)aniline (6c)

Yellow solid, Yield: 95 mg (69%) m.p 281–283 °C. ^1^H NMR (500 MHz, DMSO) δ 14.05 (s, 1H), 8.27 (d, *J* = 8.2 Hz, 1H), 7.83 (dd, *J* = 8.0, 1.3 Hz, 1H), 7.73 (t, *J* = 8.5 Hz, 1H), 7.54 (dd, *J* = 8.2, 7.1 Hz, 1H), 7.40 (dd, *J* = 7.9, 7.2 Hz, 1H), 7.35–7.29 (m, 1H), 6.96 (d, *J* = 8.0 Hz, 1H), 6.85 (s, 2H), 6.75 (t, *J* = 7.5 Hz, 1H). ^13 ^C NMR (126 MHz, DMSO) δ 164.03, 158.68, 148.42, 141.46, 133.08, 130.12, 128.06, 127.81, 123.35, 121.28, 121.09, 116.48, 116.20, 111.64, and 104.36. HRMS (ESI) *m/z*: [M + H]^+^ calculated for C_15_H_12_N_5_O 278.1036, found 278.1038.

#### 4-Fluoro-2-(5-(pyridin-4-yl)-1,3,4-oxadiazol-2-yl)aniline (6d)

Yellow solid, Yield: 99 mg (77%), m.p 226–227.5 °C. ^1^H NMR (500 MHz, DMSO) δ 8.86 (dd, *J* = 4.4, 1.6 Hz, 2H), 8.12 (dd, *J* = 4.4, 1.6 Hz, 2H), 7.75 (dd, *J* = 9.6, 3.0 Hz, 1H), 7.24 (ddd, *J* = 8.9, 8.4, 3.0 Hz, 1H), 6.96 (dd, *J* = 9.1, 4.8 Hz, 1H), 6.73 (s, 2H). ^13 ^C NMR (126 MHz, DMSO) δ 164.8, 161.2, 154.5, 152.6, 151.3, 145.5, 130.7, 121.2 (d, *J* = 23.7 Hz), 120.8, 118.07 (d, *J* = 7.5 Hz), 113.3 (d, *J* = 22.5 Hz), and 103.6 (d, *J* = 8.3 Hz). HRMS (ESI) *m/z*: [M + H]^+^ calculated for C_13_H_10_FN_4_O 257.0833, found 257.0837.

#### 4-Chloro-2-(5-(pyridin-4-yl)-1,3,4-oxadiazol-2-yl)aniline (6e)

Yellow solid, Yield: 115 mg (84%), m.p 208–209 °C. ^1^H NMR (500 MHz, DMSO) δ 8.86 (dd, *J* = 7.1, 2.7 Hz, 2H), 8.19–8.07 (m, 2H), 7.94 (dd, *J* = 4.9, 2.5 Hz, 1H), 7.33 (ddd, *J* = 7.2, 4.0, 2.1 Hz, 1H), 7.02–6.89 (m, 3H). ^13 ^C NMR (126 MHz, DMSO) δ 164.53, 161.34, 151.34, 147.41, 133.11, 130.77, 127.28, 120.88, 119.29, 118.35, and 105.05. HRMS (ESI) *m/z*: [M + H]^+^ calculated for C_13_H_10_ClN_4_O 273.0538, found 273.0537.

#### 4-Chloro-2-(5-(isoquinolin-3-yl)-1,3,4-oxadiazol-2-yl)aniline (6f)

Yellow solid, Yield: 119 mg (74%), m.p 213–214 °C. ^1^H NMR (500 MHz, DMSO) δ 9.52 (s, 1H), 8.88 (s, 1H), 8.27 (s, 2H), 7.90 (d, *J* = 37.9 Hz, 3H), 7.34 (s, 1H), 6.99 (s, 3H). ^13 ^C NMR (126 MHz, DMSO) δ 164.0, 162.77, 153.9, 147.3, 136.7, 135.6, 132.8, 132.2, 130.0, 129.4, 128.4, 128.1, 126.9, 121.4, 119.2, 118.3, and 105.3. HRMS (ESI) *m/z*: [M + H]^+^ calculated for C_17_H_12_ClN_4_O 323.0694, found 323.0688.

#### 2-(5-(Isoquinolin-3-yl)-1,3,4-oxadiazol-2-yl)-4-methoxyaniline (6g)

Yellow solid, Yield: 118 mg (74%), m.p 190–191 °C. ^1^H NMR (500 MHz, DMSO) δ 9.54 (s, 1H), 8.86 (s, 1H), 8.27 (dd, *J* = 17.0, 8.1 Hz, 2H), 7.94 (t, *J* = 7.5 Hz, 1H), 7.86 (t, *J* = 7.5 Hz, 1H), 7.36 (d, *J* = 2.7 Hz, 1H), 7.04 (dd, *J* = 9.0, 2.8 Hz, 1H), 6.93 (d, *J* = 9.0 Hz, 1H), 6.49 (s, 2H), 3.80 (s, 3H).^13 ^C NMR (126 MHz, DMSO) δ 165.1, 162.5, 153.8, 150.3, 143.2, 136.9, 135.6, 132.2, 130.1, 129.3, 128.4, 128.1, 122.0, 121.2, 118.2, 110.5, 104.1, and 56.2. HRMS (ESI) *m/z*: [M + H]^+^ calculated for C_18_H_15_N_4_O_2_ 319.1190, found 319.1191.

#### 4-(5-(2-Aminophenyl)-1,3,4-oxadiazol-2-yl)benzenesulfonamide (8a)

Yellow solid, Yield: 118 mg (75%), m.p 282–283 °C. ^1^H NMR (500 MHz, DMSO) δ 8.38–8.31 (m, 2H), 8.06 (d, *J* = 8.5 Hz, 2H), 7.89 (dt, *J* = 12.5, 6.3 Hz, 1H), 7.61 (s, 2H), 7.34–7.27 (m, 1H), 6.93 (t, *J* = 7.0 Hz, 1H), 6.79 (d, *J* = 13.9 Hz, 2H), 6.76–6.70 (m, 1H). ^13 ^C NMR (126 MHz, DMSO) δ 165.2, 161.6, 148.5, 147.0, 133.2, 128.4, 127.7, 127.1, 116.4, 116.0, and 104.2. HRMS (ESI) *m/z*: [M + H]^+^ calculated for C_14_H_13_N_4_O_3_S 317.0703, found 317.0710.

#### 4-(5-(2-Amino-5-methylphenyl)-1,3,4-oxadiazol-2-yl)benzenesulfonamide (8b)

Yellow solid, Yield: 117 mg (71%), m.p 278–279 °C.^1^H NMR (500 MHz, DMSO) δ 8.38–8.31 (m, 2H), 8.09–8.02 (m, 2H), 7.71 (d, *J* = 0.9 Hz, 1H), 7.59 (s, 2H), 7.14 (dt, *J* = 14.5, 7.3 Hz, 1H), 6.85 (t, *J* = 8.8 Hz, 1H), 6.59 (d, *J* = 21.4 Hz, 2H), 2.26 (s, 3H).^13 ^C NMR (126 MHz, DMSO) δ 165.3, 161.6, 147.1, 146.4, 134.4, 127.7, 127.1, 126.6, 124.6, 116.7, 104.0, and 20.3. HRMS (ESI) *m/z*: [M + H]^+^ calculated for C_14_H_13_N_4_O_3_S 331.0859, found 331.0866.

#### 4-(5-(2-Amino-5-methoxyphenyl)-1,3,4-oxadiazol-2-yl)benzenesulfonamide (8c)

Yellow solid, Yield: 130 mg (75%), m.p 253–254 °C.^1^H NMR (500 MHz, DMSO) δ 8.38 (t, *J* = 10.3 Hz, 2H), 8.09–8.01 (m, 2H), 7.59 (d, *J* = 7.1 Hz, 2H), 7.40 (d, *J* = 2.9 Hz, 1H), 7.03 (dd, *J* = 9.0, 2.9 Hz, 1H), 6.90 (dd, *J* = 13.1, 8.5 Hz, 1H), 6.44 (s, 2H), 3.78 (s, 3H). ^13 ^C NMR (126 MHz, DMSO) δ 165.1, 161.7, 150.4, 147.1, 143.3, 127.8, 127.1, 126.6, 122.10, 118.1, 110.8, 103.9, and 56.2. HRMS (ESI) *m/z*: [M + H]^+^ calculated for C_15_H_15_N_4_O_4_S 347.0809, found 347.0812.

#### 4-(5-(2-Amino-5-fluorophenyl)-1,3,4-oxadiazol-2-yl)benzenesulfonamide (8d)

Yellow solid, Yield: 117 mg (70%), m.p 290–291 °C.^1^H NMR (500 MHz, DMSO) δ 8.42–8.36 (m, 2H), 8.08–8.02 (m, 2H), 7.79–7.71 (m, 1H), 7.59 (s, 2H), 7.21 (tdd, *J* = 11.9, 8.7, 3.0 Hz, 1H), 6.95 (td, *J* = 9.4, 4.8 Hz, 1H), 6.71 (s, 2H).^13 ^C NMR (126 MHz, DMSO) δ ^13 ^C NMR (126 MHz, DMSO) δ 164.49 (s), 161.92 (s), 154.55 (s), 152.71 (s), 147.22 (s), 145.39 (s), 127.94 (s), 127.08 (s), 126.50 (s), 121.08 (d, *J* = 23.2 Hz), 118.02 (d, *J* = 7.5 Hz), 113.33 (d, *J* = 24.4 Hz), and 103.85 (d, *J* = 8.2 Hz). HRMS (ESI) *m/z*: [M + H]^+^ calculated for C_14_H_12_FN_4_O_3_S 335.0609, found 335.0621.

#### 4-(5-(2-Amino-5-chlorophenyl)-1,3,4-oxadiazol-2-yl)benzenesulfonamide (8e)

Yellow solid, Yield: 117 mg (67%), m.p 306–307 °C.^1^H NMR (500 MHz, DMSO) δ 8.40 (d, *J* = 8.4 Hz, 2H), 8.08–8.03 (m, 2H), 7.93 (t, *J* = 4.8 Hz, 1H), 7.60 (s, 2H), 7.33 (dd, *J* = 8.9, 2.4 Hz, 1H), 6.95 (dd, *J* = 17.6, 11.7 Hz, 3H).^13 ^C NMR (126 MHz, DMSO) δ 164.2, 161.9, 147.2 (d, *J* = 11.0 Hz), 132.9, 127.9, 127.1 (d, *J* = 13.3 Hz), 126.4, 119.2, 118.3, and 105.2. HRMS (ESI) *m/z*: [M + H]^+^ calculated for C_14_H_12_ClN_4_O_3_S 353.0313, found 353.0305.

#### 4-(5-(2-Amino-5-(trifluoromethoxy)phenyl)-1,3,4-oxadiazol-2-yl)benzenesulfonamide (8f)

Yellow solid, Yield: 138 mg (65%), m.p 242–243 °C.^1^H NMR (500 MHz, DMSO) δ 8.42–8.37 (m, 2H), 8.08–8.03 (m, 2H), 7.90 (d, *J* = 2.8 Hz, 1H), 7.60 (s, 2H), 7.34 (dt, *J* = 11.2, 5.6 Hz, 1H), 7.04–7.00 (m, 3H).^13 ^C NMR (126 MHz, DMSO) δ 164.1, 162.0, 147.6, 147.4 (d, *J* = 51.9 Hz), 147.2, 138.1, 128.0, 127.0, 126.8,126.4, 121.8, 121.1, 119.8, 117.7, and 104.0. HRMS (ESI) *m/z*: [M + H]^+^ calculated for C_15_H_12_F3N_4_O_3_S 401.0525, found 401.0536.

#### 4-(5-(2-Amino-3-fluorophenyl)-1,3,4-oxadiazol-2-yl)benzenesulfonamide (8g)

Yellow solid, Yield: 102 mg (61%), m.p 260–261 °C.^1^H NMR (500 MHz, DMSO) δ 8.38–8.33 (m, 2H), 8.08–8.04 (m, 2H), 7.78 (t, *J* = 6.3 Hz, 1H), 7.60 (s, 2H), 7.32 (ddd, *J* = 11.7, 7.9, 1.3 Hz, 1H), 6.79–6.72 (m, 3H).^13 ^C NMR (126 MHz, DMSO) δ 164.5 (d, *J* = 4.1 Hz), 161.9, 152.2, 150.3, 147.2, 137.1 (d, *J* = 15.6 Hz), 137.1(d, *J* = 15.6 Hz), 127.9, 127.1, 126.4, 124.0 (d, *J* = 3.4 Hz), 118.0 (d, *J* = 17.9 Hz), 115.7 (d, *J* = 7.3 Hz), and 106.8 (d, *J* = 5.6 Hz). HRMS (ESI) *m/z*: [M + H]^+^ calculated for C_14_H_12_FN_4_O_3_S 335.0609, found 335.0611.

#### 4-(5-(2-Amino-3,5-dimethylphenyl)-1,3,4-oxadiazol-2-yl)benzenesulfonamide (8h)

Yellow solid, Yield: 112 mg (65%), m.p 295–296 °C.^1^H NMR (500 MHz, DMSO) δ 8.34 (d, *J* = 7.6 Hz, 2H), 8.05 (d, *J* = 7.7 Hz, 2H), 7.60 (s, 3H), 7.07 (s, 1H), 6.44 (s, 2H), 2.22 (d, *J* = 35.1 Hz, 6H).^13 ^C NMR (126 MHz, DMSO) δ 165.6, 161.6, 147.1, 144.6, 135.2, 127.7, 127.1, 126.6, 125.7, 124.6, 123.7, 104.0, 20.3, and 18.1. HRMS (ESI) *m/z*: [M + H]^+^ calculated for C_16_H_17_N_4_O_3_S 345.1016, found 345.1012.

#### 4-(5-(2-Amino-3,5-dichlorophenyl)-1,3,4-oxadiazol-2-yl)benzenesulfonamide (8i)

Yellow solid, Yield: 114 mg (60%), m.p 284–285 °C.^1^H NMR (500 MHz, DMSO) δ 8.29–8.24 (m, 2H), 8.08–8.02 (m, 2H), 7.60 (s, 2H), 7.52 (t, *J* = 9.3 Hz, 1H), 6.85 (d, *J* = 8.5 Hz, 1H), 6.43 (s, 2H).^13 ^C NMR (126 MHz, DMSO) δ 164.1, 161.2, 147.3, 146.5, 133.22 (d, *J* = 41.0 Hz), 127.9, 127.2, 126.7, 117.5 (d, *J* = 33.0 Hz), and 106.9. HRMS (ESI) *m/z*: [M + H]^+^ calculated for C_14_H_11_Cl_2_N_4_O_3_S 384.9923, found 384.9927.

#### 3-(5-(2-Aminophenyl)-1,3,4-oxadiazol-2-yl)benzenesulfonamide (8j)

Yellow solid, Yield: 99 mg (63%), m.p 257–258 °C. ^1^H NMR (500 MHz, DMSO) δ 8.56 (s, 1H), 8.36 (d, *J* = 7.9 Hz, 1H), 8.07 (d, *J* = 7.9 Hz, 1H), 7.90–7.82 (m, 2H), 7.62 (s, 2H), 7.34–7.29 (m, 1H), 6.94 (d, *J* = 8.3 Hz, 1H), 6.81 (s, 2H), 6.73 (dd, *J* = 11.1, 4.0 Hz, 1H).^13 ^C NMR (126 MHz, DMSO) δ ^13 ^C NMR (126 MHz, DMSO) δ 165.1, 161.6, 148.4, 145.7, 133.2, 130.9, 130.1, 129.0, 128.3, 124.6, 124.0, 116.4, 116.0, and 104.2. HRMS (ESI) *m/z*: [M + H]^+^ calculated for C_14_H_13_N_4_O_3_S 317.0703, found 317.0711.

#### 3-(5-(2-Amino-5-methylphenyl)-1,3,4-oxadiazol-2-yl)benzenesulfonamide (8k)

Yellow solid, Yield: 107 mg (65%), m.p 264–265 °C. ^1^H NMR (500 MHz, DMSO) δ 8.55 (s, 1H), 8.37 (d, *J* = 7.7 Hz, 1H), 8.07 (d, *J* = 7.7 Hz, 1H), 7.85 (t, *J* = 7.8 Hz, 1H), 7.65 (d, *J* = 22.4 Hz, 3H), 7.15 (d, *J* = 8.1 Hz, 1H), 6.86 (d, *J* = 8.4 Hz, 1H), 6.62 (s, 2H), 2.27 (s, 3H).^13 ^C NMR (126 MHz, DMSO) δ 165.2, 161.5, 146.4, 145.7, 134.4, 130.8, 130.2, 129.0, 127.6, 124.6 (d, *J* = 2.3 Hz),124.0, 116.7, 104.0, and 20.3. HRMS (ESI) *m/z*: [M + H]^+^ calculated for C_15_H_15_N_4_O_3_S 331.0859, found 331.0855.

#### 3-(5-(2-Amino-5-methoxyphenyl)-1,3,4-oxadiazol-2-yl)benzenesulfonamide (8l)

Yellow solid, Yield: 119 mg (69%), m.p 240–242 °C. ^1^H NMR (500 MHz, DMSO) δ 8.57 (t, *J* = 1.5 Hz, 1H), 8.42–8.37 (m, 1H), 8.09–8.04 (m, 1H), 7.85 (t, *J* = 7.8 Hz, 1H), 7.64 (s, 2H), 7.38 (d, *J* = 2.9 Hz, 1H), 7.04 (dd, *J* = 9.0, 2.9 Hz, 1H), 6.92 (d, *J* = 9.0 Hz, 1H), 6.45 (s, 2H), 3.78 (s, 3H).^13 ^C NMR (126 MHz, DMSO) δ 165.0, 161.6, 150.36, 145.7, 143.2, 130.8, 130.3, 129.0, 124.5, 124.0, 121.9, 118.1, 110.9, 103.9, and 56.2. HRMS (ESI) *m/z*: [M + H]^+^ calculated for C_15_H_15_N_4_O_4_S347.0809, found 347.0818.

#### 3-(5-(2-Amino-5-fluorophenyl)-1,3,4-oxadiazol-2-yl)benzenesulfonamide (8m)

Yellow solid, Yield: 100 mg (60%), m.p225–226 °C. ^1^H NMR (500 MHz, DMSO) δ 8.59 (t, *J* = 1.6 Hz, 1H), 8.41 (d, *J* = 7.8 Hz, 1H), 8.06 (ddd, *J* = 11.1, 6.1, 4.8 Hz, 1H), 7.85 (t, *J* = 7.8 Hz, 1H), 7.74–7.67 (m, 1H), 7.63 (s, 2H), 7.23 (td, *J* = 8.6, 3.0 Hz, 1H), 6.96 (dd, *J* = 9.1, 4.8 Hz, 1H), 6.71 (s, 2H).^13 ^C NMR (126 MHz, DMSO) δ 164.38, 161.8, 154.5, 152.7, 145.7, 145.3, 130.8, 130.3, 129.1, 124.4, 124.1, 121.1, 121.0 (d, *J* = 23.1 Hz), 120.9, 118.0 (d, *J* = 7.4 Hz), 113.3, 113.2 (d, *J* = 24.3 Hz), 113.1, and 103.8 (d, *J* = 8.2 Hz). HRMS (ESI) *m/z*: [M + H]^+^ calculated for C_14_H_12_FN_4_O_3_S335.0609, found 335.0617.

#### 3-(5-(2-Amino-5-(trifluoromethoxy)phenyl)-1,3,4-oxadiazol-2-yl)benzenesulfonamide (8n)

Yellow solid, Yield: 120 mg (60%), m.p 253–255 °C. ^1^H NMR (500 MHz, DMSO) δ 8.56 (s, 1H), 8.37 (d, *J* = 8.0 Hz, 1H), 8.07 (d, *J* = 8.3 Hz, 1H), 7.85 (t, *J* = 7.8 Hz, 1H), 7.75 (d, *J* = 8.0 Hz, 1H), 7.62 (s, 2H), 7.32 (dd, *J* = 10.7, 8.0 Hz, 1H), 6.80–6.72 (m, 3H).^13 ^C NMR (126 MHz, DMSO) δ 164.4, 161.9, 152.2, 150.3, 145.8, 137.1 (d, *J* = 15.6 Hz), 130.9, 130.3, 129.2, 124.4, 124.1, 123.9 (d, *J* = 3.2 Hz), 118.0 (d, *J* = 17.9 Hz), 115.7 (d, *J* = 7.2 Hz), and 106.8 (d, *J* = 5.4 Hz). HRMS (ESI) *m/z*: [M + H]^+^ calculated for C_15_H_12_F_3_N_4_O_4_S401.0526, found 401.0533.

#### 3-(5-(2-Amino-3-fluorophenyl)-1,3,4-ovxadiazol-2-yl)benzenesulfonamide (8o)

Yellow solid, Yield: 101 mg (61%), m.p 231–233 °C. ^1^H NMR (500 MHz, DMSO) δ 8.58 (t, *J* = 1.6 Hz, 1H), 8.45–8.40 (m, 1H), 8.10–8.06 (m, 1H), 7.85 (dd, *J* = 9.0, 6.6 Hz, 2H), 7.63 (s, 2H), 7.35 (dd, *J* = 9.1, 2.0 Hz, 1H), 7.05–7.00 (m, 3H).^13 ^C NMR (126 MHz, DMSO) δ 164.0, 161.9, 147.6, 145.7, 138.0 (d, *J* = 1.9 Hz), 130.8, 130.5, 129.2, 126.7, 124.4, 124.2 (d, *J* = 33.0 Hz), 124.1, 120.9, 117.7, and 104.09. HRMS (ESI) *m/z*: [M + H]^+^ calculated for C_14_H_12_FN_4_O_3_S335.0609, found 335.0615.

### Carbonic anhydrase inhibition assay

An SX.18 MV-R Applied Photophysics (Oxford, UK) stopped-flow instrument has been used to assay the catalytic/inhibition of various CA isozymes^24^. Phenol Red (at a concentration of 0.2 mM) has been used as indicator, working at the absorbance maximum of 557 nm, with 10 mM Hepes (pH 7.4) as buffer, 0.1 M Na_2_SO_4_ or NaClO_4_ (for maintaining constant the ionic strength; these anions are not inhibitory in the used concentration), following the CA-catalysed CO_2_ hydration reaction for a period of 5–10 s. Saturated CO_2_ solutions in water at 25 °C were used as substrate. Stock solutions of inhibitors were prepared at a concentration of 10 µM (in DMSO-water 1:1, v/v) and dilutions up to 0.01 nM done with the assay buffer mentioned above. At least seven different inhibitor concentrations have been used for measuring the inhibition constant. Inhibitor and enzyme solutions were preincubated together for 10 min at room temperature prior to assay, in order to allow for the formation of the E-I complex. Triplicate experiments were done for each inhibitor concentration, and the values reported throughout the paper are the mean of such results. The inhibition constants were obtained by non-linear least-squares methods using the Cheng–Prusoff equation, as reported earlier, and represent the mean from at least three different determinations. All CA isozymes used here were recombinant proteins obtained as reported earlier by our group^25^^,^^26^.

## Results and discussion

### Chemistry

We commenced our investigation with a reaction using an equimolar ratio of isatin and 4-methyl benzohydrazide as model substrates using molecular iodine (100 mol%) and Cs_2_CO_3_ (1.0 equiv.) in DMSO at 100 °C (Table 1). The desired product was obtained in 71% yield (Table 1, entry 1). No product was obtained in the absence of either catalyst or base which suggests that an iodine/base combination is required for the reaction to occur (Table 1, entries 2–4). Exploring the possibility for improving the reaction efficiency, the effect of other alkali metal carbonates/other bases on the reaction efficiency was then examined. The transformation underwent smoothly in the presence of K_2_CO_3_ to afford the desired product **3a** in 80% yield after 12 h (Table 1, entry 5),whereas other bases, such as Na_2_CO_3_, K_3_PO_4_, and NaHCO_3_ were found to be less effective (Table 1, entries 6–8). With an attempt to further optimise the yield of the product, we investigated the influence of various iodine reagents. TBAI, N-iodosuccinimide (NIS) and KI gave poor to moderate yields, i.e. of 18, 45, and 35%, respectively (Table 1, entries 9–11). However, phenyliodine(III) diacetate (PIDA), and hydroxy(tosyloxy)iodobenzene (HITB) did not at all lead to the formation of the desired product **3a** (Table 1, entries 12–13). Furthermore, a series of experiments were also carried out in various other solvents, such as, DMF, MeCN, THF, 1,4-dioxane, EtOH, MeOH, and H_2_O. From the obtained results, it can be seen that the use of DMSO and DMF at 120 °C gave an almost identical result, albeit with a lower yield in the latter case (Table 1, entries 14–15), whereas, MeCN, THF, 1,4-dioxane, EtOH, MeOH, and H_2_O at reflux temperatures proved to be less effective (Table 1, entries 16–21). Furthermore, the iodine loading was also investigated in this reaction, and the yields were dropped to 59 and 52 at 0.75 and 0.5 equiv., respectively (Table 1, entries 22–23) of I_2_, and to a significantly lower value of 33% at 0.2 mol equiv. of I_2_ (Table 1, entry 24). We also conducted a control experiment under nitrogen atmosphere, but the yield under these conditions was diminished to 25%. This indicated that atmospheric O_2_ played an important role in the above transformation. Surprisingly, when the same reaction was performed under microwave irradiation gave better yield of **3a** (91%) within a short span of time (40 min). Indeed, the use of microwave technology has never been mentioned in the literature for the synthesis of 2–(1,3,4-oxadiazol-2-yl)aniline derivatives up until now. Thus the foregoing experiments led to the conclusion that the conditions used under entry 25 of Table 1 are the optimal ones for the reaction and, therefore, the microwave conditions were employed subsequently for all further reactions to generate compounds **3a**–**3u**/**6a**–**6g/8a**–**o**.

**Table 1.** Optimisation of the reaction conditions for the synthesis of compound **3a**^a^.

Entry	Iodine (mol%)	Base	Solvent	Yield (**3a**)^b^ (%)
1	I_2_ (100)	Cs_2_CO_3_	DMSO	71
2	I_2_	−	DMSO	n.r^d^
3	−	Cs_2_CO_3_	DMSO	n.r^d^
4	−	−	DMSO	n.r^d^
5	I_2_ (100)	K_2_CO_3_	DMSO	80
6	I_2_ (100)	K_3_PO_4_	DMSO	52
7	I_2_ (100)	Na_2_CO_3_	DMSO	59
8	I_2_ (100)	NaHCO_3_	DMSO	56
9	TBAI (100)	K_2_CO_3_	DMSO	18
10	NIS (100)	K_2_CO_3_	DMSO	42
11	KI (100)	K_2_CO_3_	DMSO	35
12	PIDA (100)	K_2_CO_3_	DMSO	n.r^d^
13	HTIB (100)	K_2_CO_3_	DMSO	n.r^d^
14^c^	I_2_ (100)	K_2_CO_3_	DMSO	86
15^c^	I_2_ (100)	K_2_CO_3_	DMF	80
16	I_2_ (100)	K_2_CO_3_	MeCN	31
17	I_2_ (100)	K_2_CO_3_	THF	15
18	I_2_ (100)	K_2_CO_3_	1,4-dioxane	45
19	I_2_ (100)	K_2_CO_3_	EtOH	39
20	I_2_ (100)	K_2_CO_3_	MeOH	27
21	I_2_ (100)	K_2_CO_3_	H_2_O	n.r^d^
22	I_2_ (20)	K_2_CO_3_	DMSO	33
23	I_2_ (50)	K_2_CO_3_	DMSO	52
24	I_2_ (75)	K_2_CO_3_	DMSO	59
25	I_2_ (100)	K_2_CO_3_	DMSO	91^e^

^a^Standard reaction conditions: **1a** (1.0 equiv.), **2a** (1.0 equiv.), reagents (equiv.) were heated in 3 ml solvent in a sealed tube for 12 h; ^b^Isolated yields; ^c^Reaction was carried out at 120 °C; ^d^n.r.: no reaction; ^e^Reaction was carried out under microwave irradiation at 160 °C.

**Scheme 2.**
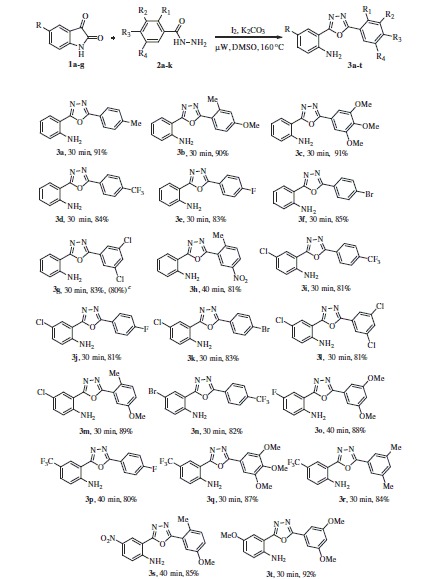
One pot synthesis of the 2-(1,3,4-oxadiazo-2-yl)aniline derivatives. (a) Reaction conditions: 1 (1 equiv.), 2 (1.05 equiv.), I_2_ (1.0 equiv.), K_2_CO_3_ (1.5 equiv.) in DMSO (3 ml) under µW irradiation at 160 °C for 30–40 min, (b) isolated yields, and (c) The reaction was performed on gram scale.

With the optimised conditions in hand, we started our exploration towards finding the potential applicability of this intramolecular decarboxylating domino reaction by attempting to prepare a variety of 1,3,4-oxadiazoles, using a varied set number of substituted isatins and benzohydrazides. The results are summarised in Scheme 2. In this way, a diversified set of 1,3,4-oxadiazoles **3a**–**3u** were obtained in moderate to excellent yields. It was found that the reactions were equally successful with both electron withdrawing (4-CF_3_, 4-F, 4-Br, and 3,5-dichloro) as well as electron donating substituents [4-Me, 2-Me-4-OMe, and 3,4,5-(OMe)_3_] on the hydrazide component. However, it may be emphasised that in contrast to other electron-withdrawing substituents, the 4-nitro group-bearing substrates required longer time to complete the reaction satisfactorily (**3h**).

**Scheme 3.**
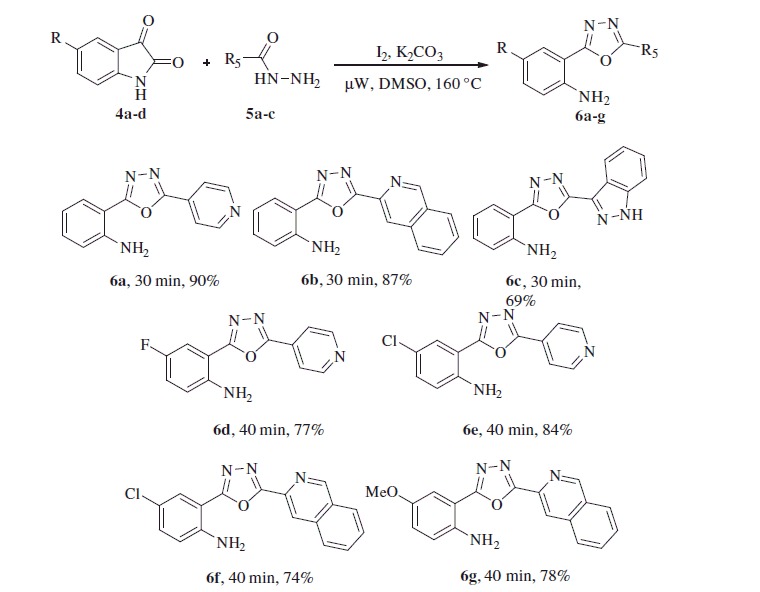
One-pot synthesis of 2-(1,3,4-oxadiazo-2-yl)aniline derivatives from various isatins and heteroaryl hydrazides. Reaction conditions: **4** (1.0 equiv.), **5** (1.05 equiv.), I_2_ (1.0 equiv.), K_2_CO_3_ (1.5 equiv.) in DMSO (3 ml) under µW irradiation at 160 °C for 30–40 min, isolated yields.

**Figure 2.**
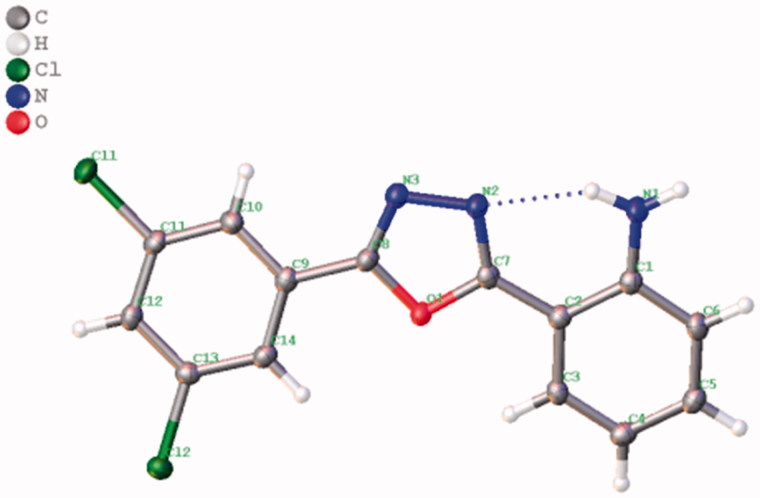
ORTEP diagram of the single crystal structure of compound **3g** as determined by X-ray crystallography.

We also studied the effect of electron-donating (–OMe) and electron-withdrawing groups (5-Cl, 5-Br, 5-F, 5-OCF_3_, and 5-NO_2_) on the isatin component on the reaction efficiency, in terms of both the yield and the reaction time. Notably, these reactions also underwent smoothly to render the corresponding 1,3,4-oxadiazoles in good to excellent yields of 80–92% (**3j**–**3u**). Considering the significance of the heterocyclic scaffolds in organic synthesis and medicinal chemistry, we further investigated as substrate of this protocol a variety of heteroaryl hydrazides, such as isonicotinoylhydrazide, isoquinoline-3-carbohydrazide, and indazole-3-carbohydrazide (Scheme 3). Under the optimal conditions mentioned above, these heteroaryl derivatives smoothly reacted with isatin and provided the corresponding 1,3,4-oxadiazoles in moderate to good yields, i.e. 69–90% (**6a**–**c**), whereas, the reactions with 5-chloro, 5-fluoro, and 5-methoxy-isatin required longer reaction times (40 min) to furnish the desired product in satisfying yields (**6d**–**g,** 74–84%). The structure of **3g** was confirmed by X-ray crystallographic analysis, as depicted in Figure 2.

**Scheme 4.**
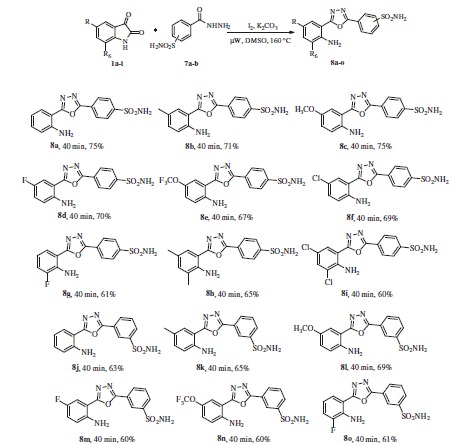
One pot synthesis of 2-(1,3,4-oxadiazolo-2-yl)aniline-benzene sulfonamide derivatives. Reaction conditions: **1** (1.0 equiv.), **7** (1.05 equiv.), I_2_ (1.0 equiv.), K_2_CO_3_ (1.5 equiv.) in DMSO (3 ml) under µW irradiation at 160 °C for 40 min, isolated yields.

In addition to various substituted aryl and heteroaryl hydrazides, we applied this protocol on hydrazides incorporating a sulfonamide moiety. Under the optimised conditions mentioned earlier, 3 or 4-sulfamoyl benzhydrazides **7a,b** reacted smoothly with a variety of substituted isatins to afford different 2-(1,3,4-oxadiazolo-2-yl)aniline-benzene sulfonamides **8a–o** (Scheme 4).

**Scheme 5.**
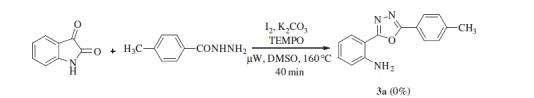
Control experiment using TEMPO.

In order to obtain some mechanistic insights into the nature of the reaction, radical trapping experiments were performed by employing TEMPO (0.5 equiv.) under the set of optimised conditions mentioned above. Indeed, no desired product was obtained when the reaction was performed in the presence of TEMPO, suggesting clearly that the reaction took place through a radical pathway (Scheme 5).

**Figure 3.**
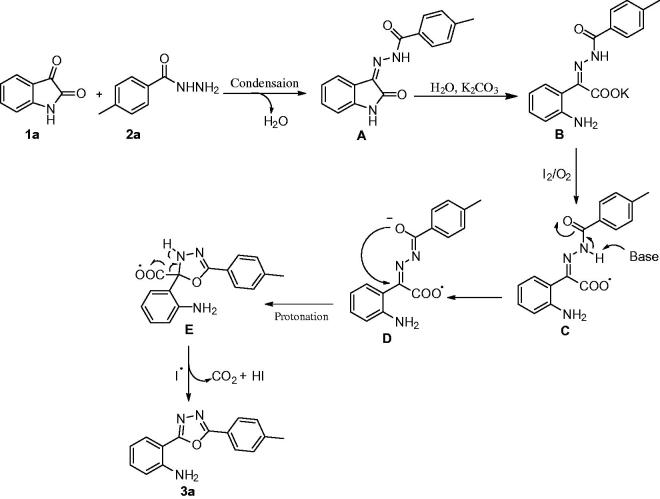
Possible reaction mechanism for the domino reaction investigated here.

In the light of the obtained results and the work reported in the literature^8^^,^^17^, a plausible reaction mechanism is proposed, which is shown in Figure 3, using **1a** and **2a** as the starting materials for the iodine-mediated domino reaction. Isatin (**1a**) condenses with the hydrazide (**2a**) to give the intermediate hydrazone **A**, which subsequently undergoes a hydrolytic ring cleavage to form the carboxylate **B**. Iodine in the presence of oxygen, oxidises **B** to form the radical intermediate **C**. Subsequently, abstraction of a proton by the base, followed by a cyclisation gave the intermediate **E**, which by elimination of one molecule of CO_2_ and HI, led to the formation of the final product **3a**.

**Figure 4.**
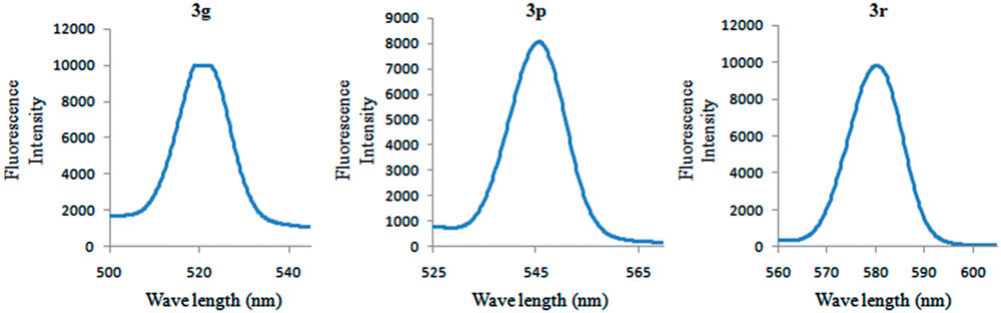
Fluorescence emission spectra of compounds **3g**, **3q**, and **3s** in DMSO.

1,3,4-Oxadiazoles are well known for exhibiting a specific fluorescence^21^. In this regard, we investigated the excitation and emission spectra of some representative molecules described here in diluted DMSO as solvent. The excitation and emission spectra of compounds **3e** and **3o** showed prominent shifts to longer wavelengths, in contrast to the other cases in which the shifts were typically only marginal. Figure 4 shows the fluorescence emission spectra of compounds **3g**, **3q**, and **3s**. Fluorescence properties of these compounds suggest that they may hold a potential for applications as chemical probes.

### Carbonic anhydrase inhibition

Carbonic anhydrases (CAs, EC 4.2.1.1) are a superfamily of metalloenzymes, present in most living organisms, in which they catalyses a simple physiological reaction, i.e. the reversible hydration of CO_2_ to bicarbonate and protons *via* a ping–pong mechanism. These enzymes are involved in many physiological and pathological processes, such as pH and CO_2_ homeostasis, respiration and transport of carbon dioxide and bicarbonate between metabolising tissues and lungs, electrolyte secretion in various tissues and organs, biosynthetic reactions (gluconeogenesis, lipogenesis, and ureagenesis); calcification, bone resorption, and tumourigenicity (in mammals)^22^^,^^23^. Dysregulated activities of these carbonic anhydrases were proven to be connected with different human diseases, and inhibition of these enzymes by small molecules represents an efficient strategy in chemotherapeutic intervention. Sulphonamides and their bioisosteres (sulfamates and sulfamides), represents the main class of pharmacologically relevant CA inhibitors. Hence, it was of interest to evaluate the CA inhibitory activity of 2-(1,3,4-oxadiazolo-2-yl)aniline-benzene sulfonamides (**8a–o**) reported here. Thus, compounds **8a–o** were investigated as inhibitors of four catalytically active human (h) CA isoforms, i. e. widespread, cytosolic, hCA I, and hCA II, the membrane-anchored hCA IV, as well as the transmembrane hCA IX, using the clinically used compound acetazolamide as a standard inhibitor.

The inhibition data are shown in Table 2. The following structure-activity relationship (SAR) can be delineated from the data of Table 2:

**Table 2.** Inhibition of hCA isoforms I, II, IV, and IX with sulphonamides **8a–o** by a stopped-flow CO_2_ hydrase assay^24^.

K_I_ (nM)*				
Compound	hCA I	hCA II	hCA IV	hCA IX
**8a**	222.2	34.1	7339.5	1892.5
**8b**	270.1	51.5	5608.9	1599.6
**8c**	320.7	16.4	5924.3	282.1
**8d**	735.5	43.7	6326.7	1604.2
**8e**	3497.2	221.5	8239.1	2366.0
**8f**	709.6	93.2	2173.5	2453.5
**8g**	81.4	6.4	2022.7	2267.5
**8h**	89.1	17.6	7592.0	2030.7
**8i**	812.6	46.4	6777.9	2738.5
**8j**	3311.9	46.9	526.0	2915.5
**8k**	5828.3	64.6	437.3	2964.0
**8l**	3514.0	515.7	588.2	2715.2
**8m**	746.6	307.0	548.3	2566.0
**8n**	344.1	480.4	9428.0	254.7
**8o**	731.0	86.3	521.5	140.3
AAZ	250	12.1	74	25.8

* Mean from three different assays, by a stopped-flow technique (errors were in the range of ±5–10% of the reported values).

- The slow cytosolic isoform hCA I was inhibited by all the examined sulphonamide derivatives **8a–o** with inhibition constants (K_I_s) spanning between 81.4 and 5828.3 nM. Sulphonamides incorporating fluoro (**8g**) and dimethylaniline moieties (**8h**) showed medium nanomolar activity (K_I_ of 81.4 and 89.1 nM, respectively). The other analogues were less potent and exhibited high nanomolar to low micromolar inhibitory potency against this isoform (K_I_s ranging between 222.2 and 5828.3, respectively)^25^^,^^26^.
- hCA II, the dominant physiological isozyme, which is an anti-glaucoma drug target, was inhibited by all the tested compounds, with efficacy spanning from the low to the high nanomolar range (K_I_s of 6.4–515.7 nM, Table 2). Among these, compound **8g** displayed the highest inhibitory activity with a K_I_ of 6.4 nM. Compounds **8a–d**, **8f**, **8h–k**, and **8o** also showed nanomolar inhibitory activity against this isoform, with K_I_s in the range of 16.4–86.3 nM, whereas remaining analogues exhibited high nanomolar inhibitory action. Among all these compounds, the 4-sulfamoyl derivatives showed a better CA II inhibitory activity compared to the 3-sulfamoyl derivatives. For example compounds, **8c** and **8g** were 31 and 13 times more potent than **8l** and **8o**, respectively. On the other hand, substitution on the aniline fragment also had a significant role on activity, i.e. *o*-fluoro substituted aniline bearing analogues **8g** and **8o** were more active in comparison to the *p*-substituted analogues **8d** and **8m**. From these observations, it can be clearly demonstrated that the substitution pattern on both phenyl rings had significant effect on the inhibitory activity against hCA II.
- hCA IV, which is a membrane-associated isoform majorly expressed in the eye, lungs, and kidneys, being involved among others in glaucoma and retinitis pigmentosa diseases, was not particularly prone to inhibition by the sulphonamides investigated here In fact, all screened molecules (**8a–o**) displayed micromolar inhibitory activity, except **8j–m** and **8o,** which showed high nanomolar CA inhibitory activity, with K_I_s of 437.3–548.3 nM (Table 2).
- hCA IX, the tumour-associated isoform, was moderately inhibited by all tested compounds with K_I_s in the range of 140.3–2964.0 nM. Substitution on both phenyl rings did not significantly influence the inhibition profile of these compounds for this isoform. Of the screened compounds, **8c**, **8n**, and **8o** exhibited better CA IX inhibitory profile against this isozyme, with K_I_s of 140.3–282.1 nM (Table 2).

## Conclusions

We have developed a more efficient and environmentally friendly protocol for the construction of 2-(1,3,4-oxadiazol-2-yl)aniline derivatives through one-pot domino decarboxylation by employing molecular iodine under microwave irradiation. This strategy works well with various substituted isatins and hydrazides belonging to both aryl and heteroaryl series, also showing good functional group tolerance. Furthermore, this protocol provided various oxadiazoles, which can be used for further functionalisation protocols. The reaction mechanism of this domino reaction was also delineated and presented in this article. Many of the synthesised molecules exhibited fluorescence properties that indicate their potential usefulness in the field of material chemistry. The synthesised 2-(1,3,4-oxadiazolo-2-yl)aniline-benzene sulfonamides (**8a–o**) were tested for their CA inhibitory activity and it was noticed that compounds **8c**, **8g,** and **8h** displayed promising and selective activity against isoform hCA II with K_I_s of 16.4, 6.4, and 17.6 nM, respectively. Such compounds may be useful for various applications in which the CA activity must be inhibited, such as for the design of anti-glaucoma, antiobesity, or antitumor agents^27^^,^^28^.
